# Improving a Web-Based Tool to Support Older Adults to Stay Independent at Home: Qualitative Study

**DOI:** 10.2196/16979

**Published:** 2020-07-22

**Authors:** Mirjam Marjolein Garvelink, Titilayo Tatiana Agbadjé, Adriana Freitas, Lysa Bergeron, Thomas Petitjean, Michèle Dugas, Louisa Blair, Patrick Archambault, Noémie Roy, Allyson Jones, France Légaré

**Affiliations:** 1 Canada Research Chair in Shared Decision Making and Knowledge Translation Laval University Québec, QC Canada; 2 Centre de recherche en santé durable (VITAM) Québec, QC Canada; 3 Centre intégré universitaire de santé et services sociaux de la Capitale-Nationale (CIUSSS-CN) Québec, QC Canada; 4 Department of Social and Preventive Medicine Faculty of Medicine Laval University Québec, QC Canada; 5 Centre for Digital Media Simon Fraser University Vancouver, BC Canada; 6 Health and Social Services Systems Knowledge Translation and Implementation Component of the Quebec SPOR SUPPORT Unit Laval University Québec, QC Canada; 7 Centre intégré de santé et de services sociaux de Chaudière-Appalaches Ste-Marie, QC Canada; 8 Centre de recherche intégré pour un système apprenant en santé et services sociaux Lévis, QC Canada; 9 Division of Critical Care Medicine Department of Anesthesiology and Critical Care Medicine Université Laval Québec, QC Canada; 10 Department of Family Medicine and Emergency Medicine Faculty of Medicine Université Laval Québec, QC Canada; 11 School of Architecture Faculty of Planning, Architecture, Arts and Design Université Laval Québec, QC Canada; 12 Department of Physical Therapy Faculty of Rehabilitation Medicine University of Alberta Edmonton, AB Canada

**Keywords:** internet-based intervention, frail elderly, caregivers, decision making, personal autonomy, housing for the elderly

## Abstract

**Background:**

Older adults desire to stay independent at home for as long as possible. We developed an interactive website to inform older adults and caregivers about ways to achieve this.

**Objective:**

This study aimed to perform an in-depth exploration among potential end users about how to improve the interactive website to better inform older adults and caregivers about ways to stay independent at home.

**Methods:**

To complement the results of a quantitative survey on the usability and acceptability of the website before implementation, we conducted a qualitative descriptive study. Using multiple recruitment strategies, we recruited a purposeful sample of older adults (aged ≥65 years) and caregivers of older adults struggling to stay independent at home. We conducted face-to-face or telephonic interviews in either English or French. In addition, we collected sociodemographic characteristics, other characteristics of participants (eg, health, digital profile, and perception of retirement homes), and experiences with using the website (factors facilitating the use of the website, barriers to its use, and suggestions for improvement). Interviews were audio recorded, transcribed verbatim, and thematically analyzed by two researchers.

**Results:**

We recruited 15 participants, including 5 older adults (mean age 75 years, SD 6) and 10 caregivers (mean age 57 years, SD 14). The mean interview time was 32 min (SD 14). Most older adults had either mobility or health problems or both, and many of them were receiving home care services (eg, blood pressure measurement and body care). Overall, participants found the website easy to navigate using a computer, reassuring, and useful for obtaining information. Barriers were related to navigation (eg, difficult to navigate with a cellphone), relevance (eg, no specific section for caregivers), realism (eg, some resources presented are not state funded), understandability (eg, the actors’ accents were difficult to understand), and accessibility (eg, not adapted for low digital literacy). Suggestions for improvement included a needs assessment section to direct users to the support appropriate to their needs, addition of information about moving into residential care, a section for caregivers, distinction between state-provided and private support services, simpler language, expansion of content to be relevant to all of Canada, and video subtitles for the hearing impaired.

**Conclusions:**

Users provided a wealth of information about the needs of older adults who were facing a loss of autonomy and about what such a website could usefully provide. The request for less generic and more personalized information reflects the wide range of needs that electronic health innovations, such as our interactive website, need to address. After integrating the changes suggested, the new website—Support for Older Adults to Stay Independent at Home (SUSTAIN)—will be implemented and made available to better assist older adults and caregivers in staying independent at home.

## Introduction

### Background

Worldwide, the proportion of older adults is increasing dramatically. In Canada, the number of adults aged 65 years and older is 17.5% of the population (2019), and by 2031, it will represent 22.7% of the population [1]. Currently, 92% of all Canadian adults aged 65 years and older live in private households [2], and most of them desire to stay independent at home for as long as possible. Studies have shown, however, that as age increases, functional and cognitive impairment, the presence of chronic diseases, a diminishing social network, and a low level of physical activity make staying at home very difficult [3-6]. As a consequence, at some point, older adults and their caregivers may face the decision about whether to remain at home (with or without assistance) or move to another location that better meets either their physical or social needs or both.

Multiple options for supporting older adults to remain independent at home are currently available [7-9]. For instance, regular home care or home visiting promotes health and delivers preventive care to older adults [8]. However, older adults and their caregivers are not always aware of these options [10,11]. This lack of knowledge can result in an hasty decision to relocate older adults, for example, to a nursing home. It is important that older adults be aware of all the options available to them—the advantages and disadvantages associated with each option—and that they can weigh up all this information and make informed decisions according to what matters most to them [12]. Research has shown that good levels of knowledge about services and support, as well as convenient housing, are associated with the likelihood of continuing to live in the community [13].

Technology can provide an easy and fast way to gain access to this information. In a previous study [14], we used a user-centered design with older adults, caregivers, and health professionals to develop an interactive decision support website called *Supporting Seniors and Caregivers to Stay Mobile at Home* (SPINACH) for older adults, caregivers, and health professionals in two Canadian provinces (Quebec and Alberta). We define *interactive* as providing a 2-way information flow between the user and the site [15]. The SPINACH website consists of 3 web pages: a home page, a video page, and a resource page [16]. On the home page, visitors have the option of selecting a language (English or French) and then the option of choosing whether to consult the video page or the resource page. The video page provides information on how different providers (1 video per provider type) can help older adults to stay independent at home. The resource page provides additional information (in text form) for staying independent at home [14]. Users can either watch only the videos relevant to their current needs or watch others if their needs change over time. They can also submit comments and information. According to an earlier quantitative usability survey, the website was deemed acceptable and potentially helpful for all kinds of end users [14]. However, it also required modifications, and we sought further insights from users as to what these might entail. In this study, we aimed to complement our quantitative survey results with a qualitative usability study to explore in-depth views of potential end users on how to improve the website, renamed Support for Older Adults to Stay Independent at Home (SUSTAIN), before its implementation.

## Methods

### Research Team

Our team is a multidisciplinary group of experts in shared decision making, primary care, rehabilitation, architecture, intensive care, and caregiving. We have been working together for 6 years on research studies aiming to develop tools and strategies to facilitate the engagement of older adults and caregivers in shared decision-making processes related to housing decisions [13,14,17-21].

### Study Design and Context

We conducted a qualitative descriptive study to improve the content of an interactive website [14,22]. Initially, our study focused on 2 English-speaking provinces in western Canada (British Columbia and Alberta) and 2 provinces in eastern Canada (Ontario [English speaking] and Quebec [French speaking]). Due to recruiting difficulties within the allotted time, we extended our recruitment to another country (France) where older adults face similar issues regarding remaining independent at home [23,24]. Although the resource information referred to resources in Canada, we believed that the French participants could provide useful perspectives on the general usability of the website in a broader range of contexts. This study was approved by the Centre Hospitalier Universitaire de Quebec—Université Laval Ethics Committee (no 2018-3751) and the University of Alberta Health Research Ethics Board (Pro00055678). We used the Consolidated Criteria for Reporting Qualitative Research checklist [25] to report the findings.

### Participants

#### Eligibility Criteria

Older adults were eligible to participate if they were aged 65 years or older and cognitively capable of indicating their informed consent to participate in an individual interview (face-to-face or over the phone). We defined *cognitively capable* as not having been diagnosed with any disorder affecting reasoning. Formal or informal caregivers were eligible to participate in individual interviews (face-to-face or telephone) if they cared for an older adult struggling to remain independent at home.

Both older adults and caregivers had to be available to consult the SPINACH website before the interview. Participants were asked to navigate through the website and to explore each section at their own pace. No minimum consultation duration was defined.

#### Recruitment and Procedures

We used multiple recruitment strategies. We contacted 4 associations of caregivers and older adults (in Quebec and Alberta) to seek their support for disseminating recruitment information. They were asked to put an advertisement on their website or in their newsletter. One association never responded, 2 promised their support but did not follow through, and the fourth association posted the information on their website newsletter but without success. Finally, because of time constraints, we opted for recruiting through the social and professional networks of our research team and the snowballing method. Persons interested in participating in the study gave their first and last name, email, and telephone number to the person who recruited them, who, in turn, forwarded it to the project coordinator. One of the trained research assistants was then assigned to follow up with the participants by evaluating their eligibility and availability for the interview. The research assistant then emailed participants the link to the SPINACH website and instructed them to explore the website and watch the videos at their own pace and convenience at least one week before the interview date. We stressed the need to consult the website before the interview, whose purpose was to capture their experience of the SPINACH website and ask for suggestions for improving it. All participants were informed that they would receive financial compensation of Can $20 (US $14.89).

#### Sample Size

We recruited a purposeful sample of caregivers and older adults [26]. Guided by the model of information power, suggesting factors to be taken into account for sample size determination, we assumed that a small number of participants were needed to reach saturation for this study because (1) the aim of this study concerned a specific experience (of consultation and navigation on the SPINACH website) and participants with specific characteristics; (2) the interviewer was experienced and knew the website well, predicting a high quality of dialog; and (3) we planned to perform an in-depth exploration of narratives [27]. Other qualitative studies conducted in the older adult population relating to the decision to relocate [28], the opportunity to make independent decisions [29], and the use of a networking website [30] conducted their studies with relatively small samples (11, 12, and 6 participants, respectively) of the population of interest. Furthermore, using data from a study involving 60 in-depth interviews, Guest et al [31] found that data saturation occurred within the first 12 interviews, but the basic elements for meta-themes were present in as early as 6 interviews. Thus, we planned to recruit at least 12 participants, while making sure to reach data saturation.

### Data Collection

We collected data through individual interviews (face-to-face or over the phone) from October 2017 to January 2018. Exceptionally, we conducted 2 dyadic interviews: (1) an older adult and his caregiver, in this case, his son, and (2) a couple, both of whom were caregivers who wished to be interviewed together. Face-to-face interviews were conducted in a room provided for this purpose in our research center or in a nearby affiliated center. Participants signed an informed consent form before the interview. Interviews were conducted by a trained female research assistant (MD, MSc in Public Health) and a trained male research assistant (TP, Master in Digital Media). Interviewers had no personal attachment to the SPINACH website and were open to all comments. We conducted the interviews in English or French, according to the preference of the participant. After each interview, the interviewers reported back to the coordinator with supporting field notes. On the basis of this, they decided whether data saturation had been reached [31].

At the beginning of the interview, the research assistant greeted the participant, introduced himself/herself, and reiterated the objectives of the interview. Each participant completed an individual sociodemographic questionnaire (Table 1). The interview grid was based on the results of a previous survey of end users on the usability and acceptability of the website [14], that is, the survey results provided the hypotheses regarding what elements of usability and acceptability should be further explored. The interview began by asking participants about their health profile, digital profile, sources of health information, and perceptions of long-term care facilities. After this, the topics discussed were related to their experience with the website and suggestions for improving it. Interviews were audio recorded with the consent of participants. The interviewer took notes on any additional relevant remarks made during each encounter.

**Table 1:** Characteristics of end users (N=15).

Characteristics			Values
**Caregivers, n (%)**			10 (67)
	**Age (years)**		
		Mean (SD)	56.9 (14)
		Range	37-70
	**Sex, n (%)**		
		Female	6 (60)
		Male	4 (40)
	**Education, n (%)**		
		University degree	10 (100)
	**Relationship with the older adult, n (%)**		
		Son/daughter	8 (80)
		Spouse	1 (10)
		Other	1 (10)
**Older adults, n (%)**			5 (34)
	**Age (years)**		
		Mean (SD)	74.6 (6)
		Range	66-83
	**Sex, n (%)**		
		Female	4 (80)
		Male	1 (20)
	**Education, n (%)**		
		High school diploma	1 (20)
		College diploma	1 (20)
		University degree	3 (60)
**Older adults including those cared for by participating caregivers, n (%)**			
	**Region/country**		
		Western Canada	4 (27)
		Eastern Canada	9 (60)
		Europe (France)	2 (13)
	**Setting**		
		Urban	9 (60)
		Semiurban	2 (13)
		Rural	4 (27)
	**Housing situation**		
		Home without home care	8 (53)
		Home with medical home care	3 (20)
		Private residence with services	4 (27)
	**Are facing a housing decision?**		
		Yes	5 (33)
		No (the decision is already made)	5 (33)
		No	4 (27)
		Maybe	1 (7)

### Data Analysis

Interviews were audio recorded and transcribed verbatim. The interview grid, based on the earlier survey results, provided the initial nodes. Overall, 2 authors independently performed deductive thematic analyses of verbatim transcripts [26,32] using qualitative data analysis software (NVivo version 12). First, the authors independently read the transcripts to familiarize themselves with the data. Each analyst proceeded with individual coding by refining and developing the pre-established nodes (including subnodes and node formulations). Afterward, coders met for a consensus meeting for 3 hours to cross-check their coding, analyze the nodes and the links between them, and categorize them. Discrepancies were discussed and resolved. The authors produced a report of relevant themes from the analysis and related quotations (Table 2 and Multimedia Appendix 1). Data saturation was reached for the presented themes.

**Table 2:** Factors facilitating the use of the website and illustrative quotations.

Theme and facilitators		Quotations
**Navigation**		
	Navigation easy	“Oh. Piece of cake, really nice, it’s really clear, big obvious menus. Cause in some you really have to hunt for the link you want.” (Caregiver 8)
	Information easy to find	“I found it useful, it was easy to manage, to find information, I would say.” (Caregiver 9)
**Relevance**		
	Helpful for decision making about housing	“It offers you a lot of links and people that you can talk to, to make the decision, because it’s a difficult one. You know you’d be happy in your own home, but you’re not safe there.” (Caregiver 8)
	Increases knowledge and potential for more	“The more relevant information you add that responds to people’s immediate needs, the more useful it will be. I learned new things and I consider myself relatively educated.” (Caregiver 5)^a^
	Reassurance (about doing the right thing)	“To have the support of somebody else saying, yes you’re doing the right thing and giving you places to look and people to talk to and that support you. Well it would have been really helpful for me.” (Caregiver 8)
	Reassurance (about others experiencing the same thing)	“It was nice, the comments that made you feel less guilty, like you can get tired of doing the cooking or that there are incontinence problems, it’s normal. Everyone has those problems. It’s a good way to reassure people.” (Caregiver 5)^a^
**Understandability**		
	Simple language	“I didn’t find that very complicated. No.” (Caregiver 2)^a^
	Transcripts useful for the hearing impaired	“What I also found useful was that there were transcripts, you could see the video, so you can also read... because some people have hearing problems or they’re not able to understand, they can read it as well.” (Caregiver 9)
	Interactive (videos)	“The interactive part like that, with the little videos—that draws people in...I’d never seen that before.” (Caregiver 1)^a^
**Realism**		
	True to life even in another country	“I’m in a French context but things are quite similar to what you have in Canada. The type of resources, the needs, it’s the same. The organizations aren’t exactly the same. But what we’re looking for is the same, i.e. the help in the medical sector and all the more social things, like meals, home help, presence at home etc.; it’s all there.” (Caregiver 1)^a^

^a^Original in French.

## Results

### Quantifiers

We reported data using the graded quantifiers *few*, *some*, *many,* and *most* [33]. On the basis of a study by Chang et al [34], we used *few* when 1 or 2 participants commented on a theme, *some* when 3 to 5 commented, *many* when 6 to 9 commented, and *most* when 9 to 15 participants commented.

### Sociodemographic Characteristics of the Participants

Between October 2017 and January 2018, we interviewed 15 end users: 60% (n=9/15) from eastern Canada, 27% (n=4/15) from western Canada, and 13% (n=2/15) from France. Two-thirds (n=10/15, 67%) of the participants were caregivers and 34% (n=5/15) were older adults. The mean interview time was 32 minutes and 35 seconds (SD 14).

Of the 5 older adults participating in the study, 60% (n=3/5) had a university degree with an average age of 75 years (SD 6). Most were living in urban areas (n=3/5, 60%,) and at home (n=4/5, 80%,). Of the 10 caregivers participating in the study, 60% (n=6/10) were women, with an average age of 57 years (SD 14); all were highly educated (n=10); and approximately 90% (n=9/10) were natural caregivers taking care of their parent (n=8) or their spouse (n=1).

Furthermore, 4 participants (27%) stated that a decision about whether to stay living at home or move to another place had already been made, whereas 5 participants (33%) were expecting to make this decision in the near future and one participant (7%) was possibly facing a housing decision (Table 1).

### Other Characteristics of the Participants

#### Health Profile of Older Adults

Most older adults involved in this study, including those cared for by a participant caregiver, were currently living at home. Many older adults were receiving general home care services (eg, body care, walking aid, and grocery shopping aid). Some of them were receiving medical home care services (eg, blood samples, medication aid, and blood pressure measurement). Most older adults had either mobility or health problems or both. Some of them had started thinking about moving to another place because of autonomy loss. Some of them were experiencing difficulties related to this decision, for example:

I looked into it...I have a friend whose mother has been placed in a residence, and she told me what she had to go through and it sounds like a nightmare.

Caregiver 6

#### Digital Profiles of Participants

The most commonly used digital devices among participants were, in order of importance, computers, cellphones, and iPads. The search engine that they used most was Google. Some participants (whether older adults or caregivers) reported using these technologies daily. At the same time, a few participants reported not being comfortable with new technologies, for example:

A lot of seniors don’t own a computer here, because it’s like... why should we?...we’ve done our thing...we’re old, we want to talk to people, you know.

Older adult 5

#### Sources of Information and Perceptions of Retirement Homes

Participants said that their main sources of information about options for staying independent at home or moving to a nursing home were, in order of importance, (1) local resources, such as community centers and health and social services; (2) their personal social network; (3) the internet; and (4) their health providers. Some participants had a negative impression of nursing homes. Negative impressions were linked to high costs, isolation, a restricted social environment, accounts of abuse in nursing homes, and loss of one’s health care team, for example:

Some family physicians will no longer see a patient after they transfer to long term care...you know you’re going to a new environment, but also your traditional healthcare team goes away too.

Caregiver 10

### Experiences With the Website

#### Factors Facilitating the Use of the Website

Overall, many participants found the website helpful (eg, diversity of resources available with their contact information) for obtaining information about how to stay independent at home. Many participants found the website acceptable in terms of the content, especially the videos. They clarified that the presentation of the various scenarios in the videos was creative, reassuring (allowed them to recognize their own situation through the scenarios and the experience sharing), and helpful for understanding the roles of the various people who can help them. Many participants liked the length of the videos and found them a good way to present information. The participants mentioned specific aspects of the website that they liked, such as its clarity, its interactivity, the diversity of the resource people, and the ease of understanding the information. Some of them found the website easy to navigate when using a computer. Participants appreciated having the transcripts of the videos on the website, which they considered especially useful for people with hearing impairment (Table 2).

#### Factors Hindering the Use of the Website

Participants also discussed factors that could limit or hinder the use of the website (Multimedia Appendix 1). These factors and their solutions generally fell into the following categories: (1) navigation (eg, difficulties using a cellphone); (2) relevance (eg, insufficient information for caregivers or about cognitive impairment); (3) interactivity (eg, out-of-date information and dead links); (4) realism (eg, lack of ethnic diversity among actors); (5) understandability (eg, print too small and language too complex); (6) accessibility (eg, unwillingness to use computers); and (7) esthetics (eg, unattractive website design).

#### Proposed Modifications to the SPINACH Website

Participants proposed several improvements that could be made to the website for each of the categories. They suggested simplifying instructions on how to use the website. They suggested adding information for those deciding about a move to a nursing home, a specific section relevant to caregivers, information relevant to people with increasing cognitive impairment, and information about safety (eg, resources for people experiencing elder abuse). They also suggested better differentiation between public and private resources, more ethnic diversity among actors, shortening the videos, and adding subtitles for the hearing impaired. Full details of the barriers and proposed modifications with illustrative quotations are presented in Multimedia Appendix 1.

Other themes that emerged from the interviews were related to the types of care received at home (general and medical), the home care equipment used, and community resources available and used.

## Discussion

### Principal Findings

Aiming to improve an interactive website for older adults and caregivers developed in a previous study [14], we asked potential end users for in-depth feedback on how to improve the website to better address their needs related to staying independent at home. Overall, participants rated the SPINACH interactive website as a useful tool for helping them obtain information about options for staying independent at home. They also listed barriers to using the website (eg, information too generic and lack of a specific section for caregivers) and made several suggestions for improving its content. These results lead us to make the following observations.

### Tailored Information

First, participants showed great interest in having tailored information. For instance, they wanted the option of specifying their city and province (eg, on a map) so that resources could be suggested based on their place of residence. This is consistent with other findings that older adults and caregivers want a personalized and flexible approach to their care (or the care of their loved ones) and their decision-making process, one that respects them as individuals [35] and provides support to help them prioritize the needs associated with their multiple conditions [36]. Older adults’ autonomy changes over time [17,19], and thus, their need for information changes too. Indeed, in line with previous research, although the SPINACH website focuses on the many options for staying independent at home, some of our participants requested more information about moving to a nursing home [37]. A decision support tool has been developed specifically to support older adults in the decision-making process about housing options [18], which could be integrated into the website to meet the needs of older adults making other choices than to stay at home.

### Supporting Caregivers

Second, the website does not yet have a specific section for caregivers. Although caregiver participants found the website helpful, they also wanted to know how to find the help they might need for themselves when caring for an older adult losing autonomy. They were also interested in knowing how to manage difficult conversations with their loved ones. This confirms the results of a recent study showing that it is difficult for caregivers to find a balance between the needs of their loved ones and their own needs [38]. In fact, this factor is associated with the burden of care felt by family caregivers caring for an older adult facing housing decisions [39]. According to a literature review about caregiver involvement in any decision with their loved one, caregivers often feel uninformed and unsupported in making informed decisions congruent with their personal values, which leads to negative feelings after decision making [40]. Moreover, a recent study showed that caregivers experience more decisional conflict than their loved ones and the same level of decisional regret [21]. Thus, creating a section for caregivers on the website with information to help them face the challenges of caregiving (eg, better address the decision-making needs of their loved ones), including navigating the health care system to get the help they need [41], will reduce the risk of decisional conflict and regret. This will also indirectly help older adults get the care they need, especially if they are cognitively impaired.

### The Challenge of Balancing Tailoring With Scaling Up

Third, participants’ comments raised interesting issues relevant to the challenges of scaling up. Although wanting the information to be more tailored, they also suggested expanding the website to be relevant to a wider population, for example, different user groups in different geographical areas, and to different decision-making needs. The unexpected French participants in this study helped us understand that French older adults and caregivers face challenges similar to those faced by Canadian older adults and caregivers in terms of housing decision making and homecare. This finding as well as the growing potential of health care technologies to deal with complex choices suggest that our platform could be scaled up to the rest of Canada and other developed countries with aging populations [42]. In the future, we plan to improve the SPINACH website in line with participants’ comments. We also plan to integrate a GPS tracker system that will provide real-time information on older adults’ outdoor mobility to promote self-management and to support them in staying at home for as long as possible. In addition, the website could further respond to users’ concerns by integrating collaborative writing apps for adding regional sources [43]. Other methods, such as algorithms for self-assessment that direct users to appropriate resources, and assigning a webmaster to update the website weekly, could also be explored.

### Limitations

Our study has some limitations. We used various strategies to recruit participants; however, our sample size was small, and some populations were not well represented, notably those with lower education levels and older adults with cognitive impairment, who could have provided a different perspective for the future format of the website. Some interviews were interrupted, and we had to contact the participants again. In addition, the sound quality was sometimes poor when interviews had to be conducted over the telephone, which had an impact on the quality of the interview. However, field notes were taken by the interviewers, which filled the gaps. Finally, we did not specify a minimum consultation time for the website or track the time participants spent on the website, so it is possible that some participants did not consult every section of the site, which may have affected their evaluation.

### Conclusions

We consulted end users to improve SPINACH, an interactive website for older adults and caregivers about options for staying independent at home [14]. Users provided a wealth of information on what such a website could usefully provide. The request for less generic and more personalized information reflects the wide range of needs such a website needs to address and raises the technological issues about how to achieve this in all electronic health innovations. Requests for more information for caregivers reflect the key role of caregivers in sustaining older people independently at home. Suggestions for more information on residential care suggest that needs can change quickly and that people prefer to be informed about the full range of options. Once the suggested changes have been made, the new SUSTAIN website will be implemented in Canada. Subsequently, a thorough analysis of the scalability of this innovation is required before it can be adapted to other contexts and cultures.

## Appendix

Multimedia Appendix 1 Barriers to use of the website, suggestions for improvement, and illustrative quotations.

## Abbreviations

SPINACH: Supporting Seniors and Caregivers to Stay Mobile at Home
SUSTAIN: Support for Older Adults to Stay Independent at Home
