# Supplementary material for: Improving a Web-Based Tool to Support Older Adults to Stay Independent at Home: Qualitative Study
Source: JMIR Mhealth Uhealth. 2020 Jul 22;8(7):e16979. doi: 10.2196/16979 (PMC7407259; doi:10.2196/16979)
Supplement: Multimedia Appendix 1 [file mhealth_v8i7e16979_app1.docx]

**Multimedia Appendix 1.** Barriers to use of the website, suggestions for improvement, and illustrative quotations.

| Theme and barriers mentioned | | Improvements (stated or implied) | Quotations |
| --- | --- | --- | --- |
| **Navigation** | | | |
|  | **Navigation difficulties when using a cellphone** | | |
|  |  | Make it easier to navigate on all types of devices | “At the beginning I couldn’t open it on my phone, but it gave me the option to go to the web version...” (Caregiver 9) |
|  | **Difficult to navigate through the website** | | |
|  |  | Add information about how to use the website (eg, how to save a page one wishes to return to later) | “Maybe even a simple little area, to tell people how to save a page or that kind of stuff, so it’s easy to go back to...like not to have to go to a computer class, just add a little section for using it.” (Older adult 3) |
|  | **Unclear access to French/English versions and respective links** | | |
|  |  | Clarify French/English sections and link to correct language versions | “You find yourself in an English version...when you’re in the French resources section.” (Caregiver 5)^a^ |
| **Relevance** | | | |
|  | **Missing information (eg, safety concerns, older adult abuse, and financial support available)** | | |
|  |  | Add information about safety and elder abuse | “Abuse of elders is widespread and if your family is abusing you, you’ll not want to...you know if it’s your grandson, you’ll be like: oh my god, it’s my grandson, I didn’t think...but he did.” (Older adult 5) |
|  |  | Add information about safety in the home | “Like that decision coach, there, who says at the beginning, ‘Go and watch it, then when you’ve seen them all, you’ll have an idea of what’s important for you’. But it doesn’t do that. For me, what’s most important is safety in my house, if I decide to stay. There was nothing at all about that.” (Caregiver 4) |
|  |  | Add information about financial support available | “I think the vast majority [of older adults] are not at ease financially... Describing the various [tax] credits...could be a way to help people maintain themselves either in their family home or in those residences...I had no idea...with the residence that my mother ended up in, that there was a tax credit.” (Caregiver 5)^a^ |
|  | **Some specifications/details are missing (eg, private vs public resources)** | | |
|  |  | Differentiate between private and public resources | “It’s really important to make a difference between what’s free and not, so people don’t get confused.” (Caregiver 6) |
|  | **Missing section (eg, a section on having difficult conversations and a specific section for caregivers)** | | |
|  |  | Add a section on having difficult conversations about safety | “It might be good if it was a resource...like how to have a difficult conversation...about safe driving or when it’s the time to give up your car keys...it would be interesting for both caregivers and health...professionals about how to have that conversation with their loved ones. Sometimes some people...they mean well, but they don’t necessarily know how to ask the question in a respectful manner. Sometimes you can ask a question, and it just shuts everything down.” (Caregiver 9) |
|  |  | Add a section for caregivers | “The way I see it...it would need...some more resources for caregivers, a little bit more information.” (Caregiver 9) |
|  |  | Personalize the website by reorganizing it according to user characteristics and health problems | “Shouldn’t you think about whether it’s a professional, a caregiver, or a senior? Don’t put them in same content, but according to who logs onto the website. And then after,...the seniors, divide them up by what illnesses they have, because some will be just old but not necessarily sick, and some will be ill. They’re not at all dealing with the same issues, or facing the same difficulties. In fact it has to be somewhat personalized.” (Caregiver 2)^a^ |
|  |  | Personalize the website according to the geographical location of the user | “I think about the resources section, they listed like all the homecare and then what are the services in Alberta and then they would list like Quebec...for me it was a little bit awkward to look at, if they’d done like a map of Canada and what you’d have to do is to click on the provinces, and then click on your city. Like it’s the way it’s sort of done now. If you’re looking for something in Calgary it might be different from Edmonton.” (Caregiver 10) |
|  | **Same information for all users/information too generic** | | |
|  |  | Personalize the support on the website by creating a needs assessment section | “For sure, for me, there is a section missing, which is a needs assessment, which should be done by the person him/herself or the caregiver who’s with us.” (Caregiver 4)^a^ |
| **Interactivity** | | | |
|  | **Out of date information and dead links** | | |
|  |  | Update information and verify links weekly | “Many of the links are inactive—you get an error message.” (Caregiver 5)^a^ |
|  |  | Messaging to let people know when there is new information | “Maybe once a month or once a week you could add more information...like every time it’s different. You know, when you see it once, well...that’s it. But if you change the information, people will keep coming back.” (Caregiver 9); “If something was added or changed on the website, you’ll get some kind of message that says, hey there’s some news, come take a look. (Older adult 3) |
| **Realism** | | | |
|  | **Some of the resource people in the videos are beyond many people’s means** | | |
|  |  | Replace with state-funded resource people, or less expensive resource people (eg. A local handyman) | “Older people would be more likely to—[if they need to] adapt where they’re living—be dealing with, like, a handyman, not an architect or a designer.” (Older adult 4) |
|  | **Does not mention waiting lists for state-funded homecare workers** | | |
|  |  | Mention getting on a waiting list as soon as possible | “You’re gonna wait a long time to have a home care worker. You’ll get a nurse to look after your wounds. But the reality... let’s say it’s not like that... It’s a shame, but the homecare worker might arrive in a year and a half, or two. There are such terrible waiting lists.” (Caregiver 4)^a^ |
|  | **Assumes everyone would prefer to stay at home** | | |
|  |  | Give information for deciding to stay home and to move to a nursing home as equal options | “People say, how to live in your home as long as possible, but none of the people say...you know...if I need to go to a retirement residence or a nursing home, like...in terms of my healthcare, that’s what I would prefer... but that isn’t really discussed that much.” (Caregiver 10) |
|  | **Lack of diversity among the actors (all white** | | |
|  |  | Ethnic diversity among actors | “The lack of diversity among the experts was quite surprising, like I know the caregiver representative...here in Ontario, it’s very common to see like a Chinese or like an Indian, for example, so...it should seem a little bit more representative, maybe including some people like that to have a better reflection of reality. Maybe just one.” (Caregiver 8) |
| **Understandability** | | | |
|  | **Accents of actors were difficult to understand** | | |
|  |  | Actors speak more slowly or hire first-language actors | “Everybody was French Canadian right? Most had very good English but there was some accents that made a few words unclear. You know, that was a problem... maybe there’s some seniors who would have had a problem catching it.” (Older adult 4) |
|  | **Complex language, jargon** | | |
|  |  | Simplify language | “You’ve got to keep it simple, because when it gets to be too much, as you get older your concentration level isn’t this good, your memory level isn’t this good, and if you have to navigate around too much and there’s too much information, you just get frustrated and then just back away. Like ‘no, no, I can’t do that’...it has to be easy to read.” (Older adult 3); “Short words, short phrases, and no jargon.” (Caregiver 4) |
|  | **Print size too small** | | |
|  |  | Increase print size | “I think the print size could be a little bit bigger, with the videos...” (Older adult 4) |
|  | **Name confusing** | | |
|  |  | Change the name of the website (SUSTAIN^b^) | “The SPINACH project, I don’t know, I don’t find [the name] very... spinach leaves? It’s not that obvious. Why SPINACH?” (Caregiver 2) |
| **Accessibility** | | | |
|  | **Website restricted to 2 provinces** | | |
|  |  | Expand website to other provinces; use map to choose relevant region | “If you had something good for all provinces...hopefully it will be across all Canada.” (Older adult 3); “If they’d done like a map of Canada and what you’d have to do is to click on the provinces, and then click on your city.” (Caregiver 9) |
|  | **Digital literacy** | | |
|  |  | Suggest health care workers bring laptop and show the website to the older adult | “I would say that it isn’t going to be used by anybody who’s 80 years and older just because, just having to use a website, like they’re not into that...My generation can use it and maybe up to 75, but I don’t think...anybody older than that would even know how to get in...So, I don’t think the website is useful for older adults.” (Caregiver 6) |
|  | **Lack of computers** | | |
|  |  | Suggest health care workers bring laptop and show the website to the older adult | “A lot of seniors don’t own a computer here, because it’s like... why should we...we’ve done our thing...we’re old, we want to talk to people, you know. Their children might...” (Older adult 5) |
|  | **Videos not adapted for hearing impaired** | | |
|  |  | Add subtitles to videos | “When you had close captioning, so that the person could read it...like on TV that might help. For people like my mother who is deaf, but she wouldn’t be watching this video anyway. People, like me, who are deaf I guess.” (Caregiver 6) |
|  | **Some videos are too long** | | |
|  |  | Shorten videos | “For somebody, maybe somebody who is ill or...at the point that they’re looking if they want to stay at home, maybe it could be short... Like for example for my mom she gets tired easily, so she can’t listen to long videos.” (Caregiver 9) |
| **Aesthetic** | | | |
|  | **Unattractive website design** | | |
|  |  | Choose more attractive colors | “It could be a bit more attractive...in terms of the background colours.” (Caregiver 1) |

^a^Original in French.

^b^SUSTAIN: SUpport for older adultS to STAy INdependent at home.
